# Differentiation of Remedies

**Published:** 1885-07

**Authors:** P. P. Wells

**Affiliations:** Brooklyn, N. Y.


### HOMŒOPATHIC PHYSICIAN.

A MONTHLY JOURNAL OF MEDICAL SCIENCE.

"If our school ever gives up the strict inductive method of Hahnemann, we are lost, and deserve only to be mentioned as a caricature in the history of medicine."—CONSTANTINE HERING.

Vol. V. JULY, 1885. No. 7.

### DIFFERENTIATION OF REMEDIES.

P. P. WELLS, M. D., BROOKLYN, N. Y.

(*Continued from page 198.*)

Acon. has of pains, etc., in the stomach, *pressure* in the epigastrium (with *fullness*), like a *weight* or a *stone*, becomes sometimes a *tension* of the *chest*, or *draws to the back*, with *sense of rigidity*, as from a *sprain ;* constant sensation as of a *cold stone* in the stomach, notwithstanding repeated vomitings and diarrhœa; painful *sense* of *swelling* in the *epigastrium*, with *loss of appetite* and *short breath*, *contraction* in *stomach* as if *from astringents; severe pains* after *eating* or *drinking;* severe *constriction*, *tension*, *pressure*, *fullness*, and *heaviness* in the *hypochondria; tense*, *painful swelling under the ribs; pushings*, (Stösse) and *pressure* in the region of the *liver*, with oppressed respiration. It will be seen these symptoms are indicative of both inflammatory and neuralgic affections. Whether the case for treatment be of the one or the other character is determined by the other and more general symptoms. If inflammatory, and the question arises—shall the case have Acon.? the period of duration since the attack was initiated becomes an important element for consideration in deciding the answer. If this be short, *i. e.*, is the inflammation in its first stage before it has resulted in deposit of its product, or when this has but slightly progressed, Acon. may be a very important if not the very best remedy. If, on the other hand, it has been of so long continuance that this process has been mostly or fully completed, Acon. is by this fact alone excluded. This principle is applicable to its use in *all* inflammatory affections. If the fibrinous deposit has been passed into the lung tissue in pneumonia, or the fibrin and serum into the pleural sack, in inflammation of these tissues, then to give Acon. is only wasting time and reducing the chances of the patient's recovery, even in cases where it may in the outset have been the best remedy and have had in it *then* the power to cut the attack short. One is not to be tempted in the later stages of these inflammations, or of any other, into giving Acon. because his patient *has fever*, and he has been told by some one who knew no better, that Acon. "cures fever." There is not even a shadow of truth in this often repeated falsehood. It cures very often fevers based on fibrinous inflammation if the symptoms of the case correspond to those of the Acon. record, and not otherwise, and always provided it be given *early enough* in the case. With other fevers it will seldom be of the least service, and should only be given to such but for the strongest reasons, founded on the similarity the law requires.

Bell. has severe pains *in the epigastrium; painless* throbbing; fullness *while stooping*, with blackness before the eyes; *contractive* pains; stitches, *cuttings*, which compel holding the breath and bending the body *backward; nocturnal*, periodical pains, with *trembling pressure*, especially *after* eating. In the epigastrium also *gnawing* or only while walking; under the *sternum*, as if from *accumulated* gas, disappears with *rumbling* in the abdomen, and increasing *nausea; tensive* pain in the evening, in bed, with distended abdomen (*upper part*); *spasms* and *cramps* in the stomach, also after every dinner; *contractive*, after every eating; *burning* in the stomach; *inflammation* of the stomach and duodenum.

Bry. has *pressure* in the stomach, especially after eating, also as if *from a* stone, with peevishness, after *eating bread;* also in the epigastrium while *walking*, immediately after *supper*, with *pressure* on the *urinary bladder* and *perineum*, disappearing while sitting; *contractive* pain in the *stomach*, especially *after eating*, followed by *cutting* in the *epigastrium, rising* of *heat, nausea*, and vomiting of food; *stitches* in the stomach while *lying on the side*, in the epigastrium when *stepping*, especially from a *false step*, or also when moving; *sore* pain in the epigastrium when *touched* or *coughing;* the *lightest* touch is unendurable; *pinching* in the epigastrium, *cuttings* as from knives; *squeezing*, with sensation of warmth and *tension* when touched; sensation of

*swelling* in the epigastrium; *burning* in the stomach or epigastrium, especially while *moving;* inflammation and cramps in the stomach, contraction of the pylorus.

In differentiating between these remedies in gastric affections, if we take *pressure*, Acon. has it in the *epigastrium* like a *weight* or *stone;* Bell. in the same locality, with *yawning*, as when walking, and *under* the *sternum*, as if *from gas*, and in the stomach after eating; Bry. has it in the stomach after eating (Bell.), and as if from a *stone* (Acon.), and *after eating bread*, which neither of the others have. It has *pressure* in the *epigastrium* while *walking* after supper, as neither of the others have, with affection of the urinary apparatus; Acon. only has sensation as if a *cold* stone were in the *stomach*, with loss of appetite; Bry. in the *epigastrium;* Acon. has *contraction* in the *stomach*, as if from *astringents;* Bell., *contractive* pains; Bry., contractive pain in *stomach* after eating; Bell. only has *painless* throbbing; Bell. has *tensive* pain, with *distended abdomen;* Bry. when *touched;* Bell. has spasms and cramps in the stomach after every noonday meal; Bry. has *squeezing*, with sensation of *warmth;* both Bell. and Bry. have *burning* in the stomach; Bry. in the epigastrium, especially while moving; Bry. only has *stitches* in the stomach while *lying on the side;* it only has *cuttings*, as if from *knives;* sore pains in the epigastrium when *touched* or when *coughing*—the *lightest* touch is unendurable; it also has *pinchings*.

In the *hypochondria*, *Acon.* alone has *severe constriction;* it has also *tension, pressure, fullness, heaviness;* Bell. has *fullness* and *heaviness*, with *throbbing* in the hepatic region; Bry. has tension in the same region, or *shooting* and *burning* from *touch, coughing*, or *breathing;* Acon. has the hepatic region painfully sensitive to *touch*, with *burning*; Bry. has stitches in the liver when touched or pressed on; Acon. has *shooting pains;* Bell. has *dull* shootings; Bry., shootings when *touched;* Bell., *cramps*, with *throbbing pains*—neither of the others have; it has *pinching* alone; Bell. has *swelling, sharp pains, throbbing, cramps*, and *deep pains* in *spleen;* Bry. has *stitches;* Acon. has *tensive, painful swelling under* the *ribs;* Bry. has *hard* swelling of the *spleen*.

These three remedies are in very important relationship to abdominal affections, especially those of an inflammatory character, though this is by no means limited to this class. Acon. has *burning* in the region of *the navel*, sometimes extending to the *epigastrium*, with anxious *throbbing* and *shooting* there, and disappearing with a *chill;* Bell. has *anxious* burning in the abdomen, and at the same time in the *chest* and *face*, with stoppage of the nose, and also with *nausea*, *great anxiety*, and sweat; Bry. has heat rising from the abdomen into the epigastrium and chest, with *heartburn;* the burning is worse at night; it is also with dry mouth and thirst.

Acon. has *pinching* in the region of the *navel*, with *grasping* and *clawing;* Bell. has *pinching* in the abdomen, compelling to sit bent forward, with *diarrhœa* and frequent *vomiting;* in the upper part of the abdomen, downward, as if in the colon; deep in the abdomen, increased by retraction of the abdominal parieties and by *bending* to the *left side;* in the *right* side of the abdomen, not permitting rising from sitting; Bry. has *pinching* around the *navel* and in the *hypogastrium*, as if after a cold, followed by diarrhœa; *constricting pinching* while *at stool.*

Acon. has *pressing together* or *squeezing* of the *navel*, then *intermittent pressure* therein, like blows; Bell. has *pressure*, as if *from a stone;* also in the evening, with *pain* in the *loins*, and as if from a *hard weight* in *walking* and *standing—not* while sitting; also *deep* in the *hypogastrium* while *lying down*, with retraction of the abdomen; Bry. has *pressure* in the *abdomen*, in the *hypogastrium*, in the region of the *navel*, with *squeezing*, or as if from a *knot*, while walking in the open air; as if from a lump in the abdomen.

Acon. alone has sensation in the *left* side, over the navel, as if a *cold* body were *pressing out.*

Acon. has *drawing* pain from *both* sides to the *navel*, and excited by *bending* the body; *drawing* from the side of the abdomen to the back, with pain in the side *when pressed;* Bell. has "*drawing* in the abdomen with *pressing* pain when *lying down;* a *dull*, irritable *drawing* in the whole circumference of the pelvis, alternately in *sacrum* and *pubes;* *drawing* in abdomen as from *flatulence*, with rumbling and passage of wind" (Encyclopædia); *drawing* pains in the abdomen, with cold feet (*Ib.*); Bry. has "drawings in both *groins*, which gradually change to *burning* on the right side." (*Ib.*)

Acon. has insupportable *cutting pains* in the abdomen, in the *morning*, in bed, with outcries, loss of self-control, and throwing himself about; Bell. has *pressing cuttings*, especially in the hypogastrium; and in the *left* side especially, and when lying on it, disappearing when lying on the *right;* in the evening before going to sleep; Bry. has severe *cuttings*, as if a *dysenteric diarrhœa* would appear, with movements in and distention of the abdomen, with subsequent diarrhœa.

Acon. has *stitches* in the side *under the ribs;* during *in-*

*spiration* in the *right* side; while *laughing loud*, in the *left*; Bell. has *stitches* in the *right* side, extending to the chest and axilla, after *clawing together* therein, while walking; *pressing stitches* about the *navel; cutting stitches* like knives between the hip and the navel, or below this; Bry., *stitches* about the navel with *twisting* pains; *cutting* stitch, extending to the stomach after drinking warm milk, compelling bending forward, and disappearing after stool.

These three remedies have *painful sensibility* to *pressure* on the abdomen. It is found in all cases of inflammation within this cavity for the treatment of which one or the other of them will be likely to be called for. With Acon. it is accompanied by *burning, tearing* pains, intensely increased by *pressure* and by turning on the *left* side; with *swollen, tense* abdomen, anxiety, hiccough, constipation, and sleeplessness. With Bell. it is as if all were excoriated and raw in the abdomen, with severe pain which permits rest nowhere; Bry. has "great *sensitiveness* of the abdomen; very *sensitive*, as if sore; tenderness of the *abdominal walls.*" (*Guiding Symptoms.*)

Acon. has *weakness* of the bowels as if from abuse of cathartic medicine, which neither of the others have.

Acon. has *swelling* of the abdomen as if from ascites; Bell. has *swelling*, with and without tension and hardness, with *constrictive* pain, with *protrusion of the colon;* Bry. has *swelling* especially *after eating*, also with *pressure* in the upper part of the abdomen; with constant movements in the abdomen as if from accumulation and constipation.

Acon. has *rattling, rumbling*, and sense of fermentation in the abdomen, sometimes with feeling of *rawness*, and sometimes only *through the night;* Bell. has *rolling, rumbling*, and *pinching* in the abdomen, with escape of abundant odorless gas; Bry. has *rumbling* and *gurgling* in the bowels; *distention* and *rumbling* especially *after eating*.

Bell. has sensation of *pressing out* of the abdominal contents when the epigastrium is pressed on, and also below the navel; neither of the others have this. It has also *spasmodic* pains, *contractive* pains, *forcing* pains, *grasping, clawing*, as if a part would be packed with nails, or as if a warp of threads gathered themselves together. Bry. has *spasmodic* pains after *eating*.

Bell. has *sensitiveness* of the abdominal *walls* to *pressure ;* Bry. has *tenderness* of abdominal *walls*, abdomen very *sensitive* as if *sore*. (Compare Hyos., Nux v., Puls., and Sulph. These are all important remedies in treating abdominal inflammations. They are all characterized by *pains* on *pressure*, and the similarity to the careless observer may be confusing. But they have differences as well, and as neither of these can do the work of the other, and as it is these differences which alone disclose to us our curative, it may help us if we remember the sensitiveness which calls for Puls. is *very superficial*, that for Sulph. but a *little less* so, while that for Nux v. is *deep* in the abdomen. The sensitiveness to pressure which calls for Hyos. seems to embrace *all* the tissues. The superficial and the deep together, and as a curative of inflammation of these, is second in importance to no other remedy.)

We are called to differentiate between these three drügs in their relations to the function of defecation oftener in treating diarrhœa and dysentery than in other diseases where the disturbance of this function may be an element of more or less importance. These diseases, oftener than otherwise, find their cure of these aberrations in other drugs related more intimately to their other symptomatic elements. Where this function is suspended (constipation), the cure is oftener effected by other drugs than either of these, and of these by Bry. oftener than by either of the other two, and of these two by Bell. oftener than by Acon.

The constipation of Acon. is characterized by *hard* stool, which is expelled by *hard* pressing; Bell., constipation with *distended* abdomen, with *heat* of the head and copious sweat, and by sense of spasmodic constriction of the sphincter ani; Bry. has *hard* stool with *pressing out* of rectum (*prolapsus*); *dry* stool, as if burnt; difficult of expulsion because *large* in size.

Acon. has diarrhœa with sweat *before* or *after* the evacuation; frequent small, soft stools, with *tenesmus* (*dysenteric diarrhœa*); diarrhœa with copious urine and pain in the abdomen; unexpected escape of thin stool when it was as if gas would pass (*see Aloes and Verat.*); watery diarrhœa; *white* stools with *red* urine; involuntary stool from paralysis of the sphincter ani.

Bell. has constant calls to stool, and often with fruitless urging or with only small discharge of diarrhœic or hard stool; tenesmus, with pressing urging to the anus and genitals, or with painful contraction of the anus; urgency, as if from diarrhœa, with heat in the abdomen; frequent small, thin stools with tenesmus *before* and *after;* diarrhœa, alternating with heat of the head; with *nausea* and pressure in the stomach; with vomiting after taking cold; vomiting after fruitless diarrhœic urgency to stool; stools are mucus, papescent, or granular and yellow; *watery* immediately after *copious sweat;* sour-smelling stools, chalky stools, *green* stools, with copious urine and sweat; before slimy stool contractive pain in the rectum and excoriating pain in upper part of the abdomen; *shuddering* at stool; no expulsive power at stool; *involuntary* stool from *paralysis* of the sphincter ani; shooting, itching, crawling in the rectum; constriction and itching of the anus; flowing hæmorrhoids.

Bry. has stool twice daily or oftener; diarrhœa from taking cold; diarrhœa *alterating* with *constipation* and *cramps* in the *stomach* every three hours, with almost *involuntary, hurried* evacuation; in the *morning* with *weakness*, compelling to lie down, or at night with *burning* in the anus; *stinking* like *rotten cheese; brown* and *thin* with *nursing children; undigested, involuntary* in *sleep; bloody, thin* stools; *before* the diarrhœa, pain in the abdomen; diarrhœa with *burning* in the *anus*, or fermentation in the abdomen; after a *hard* stool *burning* in the rectum; *itching* and *jerking stitch* upward in the anus.

Bry. is oftener found to be a curative of *constipation* than either of its other near relatives. It is to be remembered that this condition when treated by medication for its relief is always to be regarded as only *one* element in a general condition, which requires for a successful dealing with it a due consideration of *all* its other elements, or that it is oftener than otherwise that the characteristics which mark the curative for the constipation as well as for the general sick condition are found in these other and more general facts of the case.

*Urinary organs:* Acon. and Bell. have *suppression* of this secretion (*compare Iod., Secale, Strum.*, and *Tereb.*); Acon., with *pressure in* the *bladder* or *stitches in the kidneys;* Bell., with *constipation* and *copious sweat.* They have also *retention* of urine. Bry. has neither. The three have *excessive quantities* of urine. Acon., with *copious sweat* or with *diarrhœa* and *pain* in the *abdomen*, or with *distortion of the eyes* and *spasmodic contraction of the feet;* Bell. has *copious* discharge, also *at night*, and with profuse *nocturnal sweat*, with *increase of appetite* in *diarrhœa* or *coldness* of *the body;* in the *morning*, with thirst and clouded vision; Bry., with *frequent urging* and while *walking* in the *open air;* Acon. has *painful* and *anxious* urging to urinate, sometimes on *touching the abdomen*, sometimes with *copious, watery* urine; Bell. has *difficult* discharge *by drops*, and *frequent* discharge with *constant urging* of *small quantities*, or *copious*, of *pale, watery* urine; Bry. has *burning* and *cutting before* passing, and *while* passing *pain* in the *abdomen*, or sensation as if the *urethra were too narrow.*

Acon. has *difficult* discharge of *small* quantities, with *frequent*

*urging*, sometimes with *pinching* about *the navel;* while urinating sensation of *stoppage in the bladder.* This is only found with Acon. Neither of its relatives have *difficult* urination. Also Acon. alone has *copious* discharge of urine, which *after standing* deposits *blood.*

Bry. has *frequent* discharges *at night. Hurrying* urgency when the bladder is *not full*, with sensation as if the water *passed spontaneously.*

Acon. has *brown, burning* urine, with brick-colored sediment, also seldom discharge of *deep red* urine *without* sediment; Bell., yellow, turbid, clear citron color; golden or bright yellow, seldom dark or brown red; whitish; turbid as from the lees of cider or beer (hefen.), with red sediment and *white thick* sediment; Bry. has hot urine, red or brown and *scanty* and *frequent*, like *clear water.*

Acon. has *involuntary* discharge of urine from paralysis of the bladder; Bell. has *inability to* retain the urine; *involuntary* discharge from *paralysis* of the *sphincter vessicæ;* escape of urine in deep *sleep;* Bry. has involuntary escape *of drops while in motion;* escape of drops *after urinating.*

Acon. has *burning* and *tenesmus* of the *neck of the bladder* when *not* urinating; pain in the bladder while walking; Bell. has *stitch* in the *urethea*, behind the glans, also *while walking;* in the bladder *turning* and *twisting* as if from a *worm; pressure* at night; *after* urinating, *smarting* of the *edge of the prepuce; while* urinating, *drawing* in the *spermatic cord.* Bry. has *burning* (also with itching and stitches), pressure, drawing and tearing in the urethea when *not* passing water.

[TO BE CONTINUED.]

